# A novel comparative case study of entropy generation for natural convection flow of proportional-Caputo hybrid and Atangana baleanu fractional derivative

**DOI:** 10.1038/s41598-021-01946-4

**Published:** 2021-11-23

**Authors:** Dolat khan, Poom Kumam, Wiboonsak Watthayu

**Affiliations:** 1 Fixed Point Research Laboratory, Fixed Point Theory and Applications Research Group, Center of Excellence in Theoretical and Computational Science (TaCS-CoE), Faculty of Science, King Mongkut’s University of Technology Thonburi (KMUTT), 126 Pracha Uthit Rd., Bang Mod, Thung Khru, Bangkok, 10140 Thailand; 2 Center of Excellence in Theoretical and Computational Science (TaCS-CoE), Faculty of Science, Thonburi (KMUTT), King Mongkut’s University of Technology, 126 Pracha Uthit Rd., Bang Mod, Thung Khru, Bangkok, 10140 Thailand; 3 Department of Medical Research, China Medical University Hospital, China Medical University, Taichung, 40402 Taiwan

**Keywords:** Fluid dynamics, Applied mathematics

## Abstract

This article focused on the comparative study of entropy generation for natural convection flow of the newly proportional Caputo hybrid and Atangana baleanu fractional derivative. The governing equation is formed as the set of partial differential equations with the physical boundary conditions. The report of entropy generation is investigated for the first time for proportional–Caputo hybrid model and comparison are sorts out with generalized Atangana baleanu fractional derivative. The Bejan number is also compared for the mention fractional derivatives. Graphs show the impact of various factors on the minimization and maximizing of entropy production. The newly proportional Caputo hybrid operator has a good memory effect rather than Atangana baleanu fractional operator.

## Introduction

The natural generalization of classical calculus, which involves non-integer order derivatives and integrals, is fractional calculus (FC), which has been a popular and commonly used method for better modelling and regulation of processes in many fields of science and engineering in recent years. FC is needed to describe certain physical processes that have “intrinsic” fractional order descriptions^1–11^. In certain cases, FC models physical processes more accurately than traditional calculus. It has been an important instrument in many fields of physics, optics, chemistry, architecture, economics, and bioengineering after its success in describing anomalous diffusion non-integer order calculus in one and multidimensional space. Fractional order results are a powerful tool for illustrating memory and inherited properties in a variety of processes and materials^12^. Ross^13^, who organized and edited the University of New Haven's First Conference on Fractional Calculus and its Applications in June 1974, shortly after completing his Ph.D. dissertation. After at the same year Oldham and Spanier^14^ collaborate jointly and issued a book for the fractional calculus. Fractional calculus can be categorized into two major types, that is classical fractional derivatives and new fractional derivatives. Sonin–Letnikov derivative, Grünwald–Letnikov derivative, Liouville derivative, Miller–Ross derivative, Weyl derivative^15–18^, etc. are examples of classical fractional derivative. While Hilfer derivative, Katugampola derivative, Chen derivative, Davidson derivative, Atangana–Baleanu derivative, Caputo derivative and Caputo Fabrizio derivative, etc.^19–23^ are the examples of new fractional derivative. From the past two decay, the researchers are working on the new fractional derivatives as much as from the classical fractional derivative in their studies. More recently in^24–27^ apply the concept of Atangana–Baleanu derivative and Caputo Fabrizio to study the problems regarding biomathematics. In fluid dynamic Ali et al*.*^28^ investigate the influence of MHD for Walters’-B fluid model along with free convection by busing the concept of Caputo-Fabrizio derivatives. In the same year, Abro and Khan^29^ report the impact of porous medium and MHD for the Casson fluid model by applying the same idea of Caputo-Fabrizio derivatives. After this Sheikh et al*.*^30^ and^31^ add a comparative study of Caputo-Fabrizio and Atangana-Baleanu fractional derivative for Casson fluid model and application of nanofluids in the solar collector, respectively. After this Saqib et al*.*^32^ report the entropy generation of fractionalized nanofluids. And find out the exact solution along with the porous medium.

Any irreversible mass and heat transfer process, including body motion, fluid flow, heat exchange, anelastic deformation of solids, substances mixing or expanding, and any irreversible thermodynamic cycle, with thermal machines such as heat engines, power plants, heat pumps, and refrigerators produce entropy generation. The second law of thermodynamics is thought to be more appropriate for technical applications than the first. Because certain agents, such as internal pressure, vibration, rotating, and kinetic energy of molecules, allow heat energy to be lost that cannot be converted into work. Such damages in physical life cannot be recouped without any effort. These are classified as irreversibilities, and they must be decided within each framework. The dimensionless Bejan number and the entropy production rate can be used to improve the efficiency of a system. Bejann^33,34^ was the first to investigate device irreversibility impacts. He discovered that these effects are caused by three major factors: thermal, fluid inertia, and magnetic effects. By reducing irreversibilities, the best thermodynamical device designs may be accomplished. As a result, entropy optimization is thought to be the best method for assessing the system's results. We have compiled a list of useful articles on entropy production that have been scrutinized by several scholars and analysts^35–39^. More recently Mliki and Abbassi^40^ report MHD natural convection heat transfer entropy generation in a hot incinerator for hybrid nano liquid. They find the numerical results by applying the lattice Boltzmann method. Analytically and numerically, Azhar et al*.*^41^ examine the influence of the time-fractional Caputo-Fabrizio derivative on the creation of entropy in a rotating channel with the temperature of the channel walls increasing exponentially and unstable hydromagnetic fractional Couette flow. In recent decay, Anderson and Ulness^42^ present a newly proportional hybrid derivative operator as follows.

1 $${}^{p}D_{\alpha } f(t) = K_{0} (\alpha ,t)f^{\prime}(t) + K_{1} (\alpha ,t)f(t),$$

This operator appears generally in control theory, and it has to do with the broad and growing theory of conformable derivatives. Recently Baleanu et al*.*^43^ worked and present a new kind of fractional operator with power law kernel. Which is combination of proportional fractional operator and Caputo fractional operator in a single operator, known as constant proportional Caputo type fractional derivative. After this innovation many researchers are consider this new operator in their work. Asjad^44^ apply successfully this novel fractional operator in fluid dynamic problem. After that Aleem et al*.*^45^ extend this work in fluid dynamic and report heat transfer analysis of MHD Casson fluid through a porous medium with this operator. Where the Laplace transform method is used in both papers to obtain the exact solution. Further, Ahmad et al*.*^46^ applied this operator for heat and mass transfer analysis of the slippage flow of viscous fluid with single‐wall carbon nanotube. In 2021 this operator gets more attention, and many researchers are used for different studies, some of them are^47–50^. Based on this motivation and Best of the author's knowledge and in comparison, to most earlier works, this paper has the following comparative advantages:

1. In this article, we are attempting to use the concept of constant proportional Caputo type fractional derivative, to investigate a novel, general and comparative report of entropy generation for natural convection.
2. This is the first attempt to investigate the entropy generation of natural convection flow for such a hybrid fractional model and compared with Atangana baleanu fractional derivative.
3. This investigation as an application of hybrid fractional derivative in fluid mechanics.
4. The mention fractional derivatives make generalized Bejan number and entropy generation for better curve fitting to experimental data.

## Mathematical formulation

Let consider the natural convection unsteady flow of Newtonian fluid in $y_{1} > 0$ region, where the plane surface is situated at $y_{1} = 0$ with a static end at $x = 0$. Initially, the fluid and surface are not moving with a constant temperature $\Theta_{\infty }$. After a period of time $t_{1} = 0^{ + }$, the fluid temperature level is increased to $\Theta_{w}$. The equations defining the flow are approximated via the Boussinesq approximation as^45^.

2 $$\rho \frac{{\partial u\left( {y_{1} ,t_{1} } \right)}}{{\partial t_{1} }} = \mu \frac{{\partial^{2} u\left( {y_{1} ,t_{1} } \right)}}{{\partial y_{1}^{2} }} + g\left( {\rho \beta_{\Theta } } \right)\left( {\Theta \left( {y_{1} ,t} \right) - \Theta_{\infty } } \right),$$

where $u\left( {y_{1} ,t_{1} } \right)$,$\rho ,\,\,\mu ,\,\,g,\,\,$ and $\beta_{\Theta }$ are fluid velocity in x direction, density of the fluid, dynamic viscosity, gravitational acceleration and the volumetric coefficient of thermal expansion. Furthermore, the energy equation^45^ is

3 $$\rho C_{p} \frac{\partial \Theta }{{\partial t_{1} }}\left( {y_{1} ,t_{1} } \right) = K\frac{{\partial^{2} \Theta }}{{\partial y_{1}^{2} }}\left( {y_{1} ,t_{1} } \right),$$

Initial and boundary conditions are as follows:

4 $$u\left( {y_{1} ,0} \right) = 0,\;\,\Theta \left( {y_{1} ,0} \right) = \Theta_{\infty } \,\,{\text{for all}}\, \, y_{1} \ge 0,\,$$

5 $$u\left( {0,t_{1} } \right) = 0,\,\,\,\Theta \left( {0,t_{1} } \right) = \Theta_{\infty } + \left( {\Theta_{w} - \Theta_{\infty } } \right)t,\,\,t_{1} > 0,\,$$

6 $$u\left( {\infty ,t_{1} } \right) \to 0,\,\,\,\Theta \left( {\infty ,t_{1} } \right) \to \Theta_{\infty } ,\,\,t_{1} > 0.$$

The following dimensionless variables are introduced:

7 $$y_{1}^{*} = \frac{U}{{\nu_{f} }}y_{1} ,\,\;t_{1}^{*} = \frac{{U^{2} }}{{\nu_{f} }}t_{1} ,\,\;u^{*} = \frac{u}{U},\,\;\,\theta = \frac{{\Theta - \Theta_{\infty } }}{{\Theta_{w} - \Theta_{\infty } }},$$

Using Eq. (7) into Eqs. (2) and (6), we get (^*^ is dropped for simplicity)

8 $$\frac{\partial u}{{\partial t_{1} }}\left( {y_{1} ,t_{1} } \right) = \frac{{\partial^{2} u}}{{\partial y_{1}^{2} }}\left( {y_{1} ,t_{1} } \right) + Gr\theta \left( {y_{1} ,t_{1} } \right),\,$$

9 $$\frac{{\partial \theta \left( {y_{1} ,t_{1} } \right)}}{{\partial t_{1} }} = \frac{1}{\Pr }\frac{{\partial^{2} \theta \left( {y_{1} ,t_{1} } \right)}}{{\partial y_{1}^{2} }},$$

10 $$u\left( {y_{1} ,0} \right) = 0,\,\,\,\theta \left( {y_{1} ,0} \right) = 0\,\;{\text{for all }}y_{1} \ge 0,\,$$

11 $$u\left( {0,t_{1} } \right) = 0,\,\,\theta \left( {0,t_{1} } \right) = t_{1} ,\;\,t_{1} > 0,\,$$

12 $$u\left( {\infty ,t_{1} } \right) \to 0,\,\,\,\theta \left( {\infty ,t_{1} } \right) \to 0,\;\;t_{1} > 0,$$

where

$$Gr = \frac{{g\beta_{\Theta } \left( {\Theta_{w} - \Theta } \right)_{\infty } }}{{U^{3} }}\,,\,\,\Pr = \frac{{\mu C_{p} }}{K}.$$

### Solution via constant proportional Caputo fractional derivative

The proportional Caputo hybrid is define as^43^;

13 $${}^{CPC}D_{t}^{\alpha } g\left( t \right) = \frac{1}{{\Gamma \left( {1 - \alpha } \right)}}\int\limits_{0}^{t} {\left( {K_{1} (\alpha )f(\tau ) + K_{0} (\alpha )f^{\prime}(\tau )} \right)} \left( {t - \tau } \right)^{ - \alpha } d\tau ,$$

where $K_{1}$ and $K_{0}$ are functions of the variable $t$ and the parameter $\alpha \in \left[ {0,\,\,1} \right]$ which satisfy the following conditions for all $t \in {\mathbb{R}}$.

14 $$\mathop {\lim }\limits_{{\alpha \to 0^{ + } }} K_{0} \left( {\alpha ,t} \right) = 0,\,\,\,\mathop {\lim }\limits_{{\alpha \to 1^{ - } }} K_{0} \left( {\alpha ,t} \right) = 1,\,\,K_{0} \left( {\alpha ,t} \right) \ne 0,\,\,\alpha \in \left( {0,\,\,1} \right],$$

15 $$\mathop {\lim }\limits_{{\alpha \to 0^{ + } }} K_{1} \left( {\alpha ,t} \right) = 1,\,\,\,\mathop {\lim }\limits_{{\alpha \to 1^{ - } }} K_{1} \left( {\alpha ,t} \right) = 0,\,\,K_{1} \left( {\alpha ,t} \right) \ne 0,\,\,\alpha \in \left[ {0,\,\,1} \right).$$

To develop a constant proportional Caputo fractional model, the time derivative in unsteady terms of Eqs. (8) and (9).

16 $${}^{CPC}D_{t}^{\alpha } u\left( {y_{1} ,t_{1} } \right) = \frac{{\partial^{2} u}}{{\partial y_{1}^{2} }}\left( {y_{1} ,t_{1} } \right) + Gr\theta \left( {y_{1} ,t_{1} } \right),\,$$

17 $${}^{CPC}D_{t}^{\alpha } \theta \left( {y_{1} ,t_{1} } \right) = \frac{1}{\Pr }\frac{{\partial^{2} \theta \left( {y_{1} ,t_{1} } \right)}}{{\partial y_{1}^{2} }},$$

where $^{CPC}D_{t}^{\alpha } \left( \right)$ is constant proportional Caputo fractional derivative.

Taking Laplace transform of Eqs. (9)–(11) and (16)–(17). We get

18 $$\left\{ {\left( {\frac{{k_{1} \left( \alpha \right)}}{q} + k_{0} \left( \alpha \right)} \right)q^{\alpha } } \right\}\overline{u} \left( {y_{1} ,q} \right) = \frac{{\partial^{2} \overline{u} }}{{\partial y_{1}^{2} }}\left( {y_{1} ,q} \right) + Gr\overline{\theta } \left( {y_{1} ,q} \right),\,$$

19 $$\Pr \left\{ {\left( {\frac{{k_{1} \left( \alpha \right)}}{q} + k_{0} \left( \alpha \right)} \right)q^{\alpha } } \right\}\overline{\theta } \left( {y_{1} ,q} \right) = \frac{{\partial^{2} \overline{\theta }\left( {y_{1} ,q} \right)}}{{\partial y_{1}^{2} }},$$

Subject to the transform boundary conditions.

20 $$\overline{u} \left( {y_{1} ,0} \right) = 0,\,\,\,\overline{\theta } \left( {y_{1} ,0} \right) = 0\,\;{\text{for all }}y_{1} \ge 0,\,$$

21 $$\overline{u} \left( {0,q} \right) = 0,\,\,\overline{\theta } \left( {0,q} \right) = \frac{1}{{q^{2} }},\;\,q > 0,\,$$

22 $$u\left( {\infty ,t_{1} } \right) \to 0,\,\,\,\theta \left( {\infty ,t_{1} } \right) \to 0,\;\;t_{1} > 0,$$

To solve Eqs. (18) and (19) we get,

23 $$\overline{\theta } \left( {y_{1} ,q} \right) = \frac{1}{{q^{2} }}e^{{ - y_{1} \sqrt {\Pr \left( {\frac{{k_{1} \left( \alpha \right)}}{q} + k_{0} \left( \alpha \right)} \right)q^{\alpha } } }} ,$$

24 $$\overline{u} \left( {y_{1} ,q} \right) = \frac{Gr}{{(1 - \Pr )\left( {\frac{{k_{1} \left( \alpha \right)}}{q} + k_{0} \left( \alpha \right)} \right)q^{\alpha + 2} }}\left[ {e^{{ - y_{1} \sqrt {\Pr \left( {\frac{{k_{1} \left( \alpha \right)}}{q} + k_{0} \left( \alpha \right)} \right)q^{\alpha } } }} - e^{{ - y_{1} \sqrt {\left( {\frac{{k_{1} \left( \alpha \right)}}{q} + k_{0} \left( \alpha \right)} \right)q^{\alpha } } }} } \right].$$

In the more suitable form, we write as

25 $$\overline{\theta } \left( {y_{1} ,q} \right) = \sum\limits_{k = 1}^{\infty } {\sum\limits_{p = 0}^{\infty } {\frac{{\left( { - y_{1} \sqrt {\Pr } } \right)^{k} \left\{ {k_{1} \left( \alpha \right)} \right\}^{p} }}{{k!p!\left\{ {k_{1} \left( \alpha \right)} \right\}^{{p - \frac{k}{2}}} q^{{2 + p - \frac{ak}{2}}} }}} } \frac{{\Gamma \left( {\frac{k}{2} + 1} \right)}}{{\Gamma \left( {\frac{k}{2} + 1 - p} \right)}},$$

26 $$\begin{aligned} \overline{u} \left( {y_{1} ,q} \right) & = \frac{Gr}{{1 - \Pr }}\sum\limits_{k = 0}^{\infty } {\sum\limits_{p = 0}^{\infty } {\sum\limits_{r = 0}^{\infty } {\sum\limits_{s = 0}^{\infty } {\frac{{\left( { - y_{1} } \right)^{k} \left( { - 1} \right)^{r} \left\{ {k_{1} \left( \alpha \right)} \right\}^{p\, + \,s} }}{{k!p!s!\left\{ {k_{0} \left( \alpha \right)} \right\}^{{p\,\, - \,\,\frac{k}{2} + s - r}} q^{{p - \frac{\alpha k}{2} + q - \alpha r}} }}} } \frac{{\Gamma \left( {\frac{k}{2} + 1} \right)\Gamma \left( {r + 1} \right)}}{{\Gamma \left( {\frac{k}{2} + 1 - p} \right)\Gamma \left( {r - s + 1} \right)}}} } \\ & \quad - \frac{Gr}{{1 - \Pr }}\sum\limits_{k = 0}^{\infty } {\sum\limits_{p = 0}^{\infty } {\sum\limits_{r = 0}^{\infty } {\sum\limits_{s = 0}^{\infty } {\frac{{\left( { - y_{1} \sqrt {\Pr } } \right)^{k} \left( { - 1} \right)^{r} \left\{ {k_{1} \left( \alpha \right)} \right\}^{p\, + \,s} }}{{k!p!s!\left\{ {k_{0} \left( \alpha \right)} \right\}^{{p\,\, - \,\,\frac{k}{2} + s - r}} q^{{p\,\, - \,\,\frac{\alpha k}{2} + q - \alpha r}} }}} } \frac{{\Gamma \left( {\frac{k}{2} + 1} \right)\Gamma \left( {r + 1} \right)}}{{\Gamma \left( {\frac{k}{2} + 1 - p} \right)\Gamma \left( {r - s + 1} \right)}}} } . \\ \end{aligned}$$

After taking Laplace inversion we get

27 $$\theta \left( {y_{1} ,t_{1} } \right) = \sum\limits_{k = 1}^{\infty } {\sum\limits_{p = 0}^{\infty } {\frac{{\left( { - y_{1} \sqrt {\Pr } } \right)^{k} \left\{ {k_{1} \left( \alpha \right)} \right\}^{p} }}{{k!p!\left\{ {k_{1} \left( \alpha \right)} \right\}^{{p - \frac{k}{2}}} }}} } \frac{{t_{1}^{{1 + p - \frac{\alpha k}{2}}} \Gamma \left( {\frac{k}{2} + 1} \right)}}{{\Gamma \left( {2 + p - \frac{\alpha k}{2}} \right)\Gamma \left( {\frac{k}{2} + 1 - p} \right)}},$$

28 $$\begin{aligned} u\left( {y_{1} ,t_{1} } \right) & = \frac{Gr}{{1 - \Pr }}\sum\limits_{k = 0}^{\infty } {\sum\limits_{p = 0}^{\infty } {\sum\limits_{r = 0}^{\infty } {\sum\limits_{s = 0}^{\infty } {\frac{{\left( { - y_{1} } \right)^{k} \left( { - 1} \right)^{r} \left\{ {k_{1} \left( \alpha \right)} \right\}^{p\, + \,s} }}{{k!p!s!\left\{ {k_{0} \left( \alpha \right)} \right\}^{{p\,\, - \,\,\frac{k}{2} + s - r}} }}} } \frac{{t_{1}^{{p - \frac{\alpha k}{2} + q - \alpha r}} \Gamma \left( {\frac{k}{2} + 1} \right)\Gamma \left( {r + 1} \right)}}{{\Gamma \left( {\frac{k}{2} + 1 - p} \right)\Gamma \left( {r - s + 1} \right)}}} } \\ & \quad - \frac{Gr}{{1 - \Pr }}\sum\limits_{k = 0}^{\infty } {\sum\limits_{p = 0}^{\infty } {\sum\limits_{r = 0}^{\infty } {\sum\limits_{s = 0}^{\infty } {\frac{{\left( { - y_{1} \sqrt {\Pr } } \right)^{k} \left( { - 1} \right)^{r} \left\{ {k_{1} \left( \alpha \right)} \right\}^{p\, + \,s} }}{{k!p!s!\left\{ {k_{0} \left( \alpha \right)} \right\}^{{p\,\, - \,\,\frac{k}{2} + s - r}} }}} } \frac{{t_{1}^{{p - \frac{\alpha k}{2} + q - \alpha r}} \Gamma \left( {\frac{k}{2} + 1} \right)\Gamma \left( {r + 1} \right)}}{{\Gamma \left( {\frac{k}{2} + 1 - p} \right)\Gamma \left( {r - s + 1} \right)}}} } . \\ \end{aligned}$$

### Solution via Atangana–Baleanu fractional derivative without singular and local kernel

The Atangana–Baleanu fractional operator “${}^{AB}D_{t}^{\alpha } g\left( t \right)$” is based on Mettang–Leffler function without Singular and local kernel which is defined as^51^.

29 $${}^{AB}D_{t}^{\alpha } g\left( t \right) = \frac{N(\alpha )}{{\left( {1 - \alpha } \right)}}\int\limits_{0}^{t} {E_{\alpha } \left\{ {\frac{{ - \alpha (\tau - t)^{\alpha } }}{1 - \alpha }} \right\}} f^{\prime}\left( {y,\,\,t} \right)dt,$$

where $N(\alpha )$ is normalization function such that $N(0) = N(1) = 1$ and $E_{\alpha } \left\{ { - t^{\alpha } } \right\} = \sum\limits_{k = 0}^{\infty } {\frac{{( - t)^{\alpha k} }}{\Gamma (\alpha k + 1)}}$ is the Mettag–Leffler function.

To develop AB fractional model, the time derivative in unsteady terms of Eqs. (8) and (9) becomes.

30 $${}^{AB}D_{t}^{\alpha } u\left( {y_{1} ,t_{1} } \right) = \frac{{\partial^{2} u}}{{\partial y_{1}^{2} }}\left( {y_{1} ,t_{1} } \right) + Gr\theta \left( {y_{1} ,t_{1} } \right),\,$$

31 $${}^{AB}D_{t}^{\alpha } \theta \left( {y_{1} ,t_{1} } \right) = \frac{1}{\Pr }\frac{{\partial^{2} \theta \left( {y_{1} ,t_{1} } \right)}}{{\partial y_{1}^{2} }},$$

Taking Laplace transform of Eqs. (30–31), and solved we get,

32 $$\overline{\theta } \left( {y_{1} ,q} \right) = \frac{1}{{q^{2} }}e^{{ - y_{1} \sqrt {\Pr \frac{{q^{\alpha } }}{{(1 - \alpha )q^{\alpha } + \alpha }}} }} ,$$

33 $$\overline{u} \left( {y_{1} ,q} \right) = \frac{{\left\{ {(1 - \alpha )q^{\alpha } + \alpha } \right\}Gr}}{{(1 - \Pr )q^{\alpha + 2} }}\left[ {e^{{ - y_{1} \sqrt {\frac{{\Pr q^{\alpha } }}{{(1 - \alpha )q^{\alpha } + \alpha }}} }} - e^{{ - y_{1} \sqrt {\frac{{q^{\alpha } }}{{(1 - \alpha )q^{\alpha } + \alpha }}} }} } \right].$$

The inverse Laplace of Eqs. (32) and (33) is find out numerically and plotted graphically.

## Entropy or irreversibility analysis

Wastage or loss of useful power in thermodynamical systems, as well as how to limit damage, becomes an important and difficult task for scientists and engineers. For that way entropy generation is playing an important role, for our problem, it is given by:

34 $$Ns = \left( {\frac{{\partial \theta \left( {y_{1} ,t_{1} } \right)}}{{\partial y_{1} }}} \right)^{2} + \frac{Br}{\Omega }\left( {\frac{{\partial u\left( {y_{1} ,t_{1} } \right)}}{{\partial y_{1} }}} \right)^{2} ,$$

where $Br$ and $\Omega$ is the dimensionless Brinkman number and temperature difference, denoted by,

$$\,\Omega = \frac{{\Theta_{W} - \Theta_{\infty } }}{{\Theta_{\infty } }},\,\,B_{r} = \frac{\mu }{{\kappa \left( {\Theta_{W} - \Theta_{\infty } } \right)}}.$$

The expression in Eq. (34) can be written as the amount of entropy generation caused by heat transfer ($N_{H}$) and local entropy generation due to fluid friction irreversibility $N_{F}$.

$$Ns = N_{F} + N_{H} ,$$

Additionally, the Bejan number $B_{e}$ is known as,

35 $$B_{e} = \frac{{N_{H} }}{Ns}.$$

Bejan number has a significant reputation to give an idea that is affected because of fluid fraction and magnetic field control over the heat transfer. “

## Graphical results and discussion

This work focused on the application of new developed fractional derivative that is proportional Caputo hybrid derivative for entropy generation natural in convection flow through a vertical plate. Furthermore, the results are compared with Atangana baleanu fractional derivative graphically. The existing model is converted to a proportional Caputo hybrid derivative model and solve via Laplace transform technique. Mathcad-15 software was used to generate some numerical simultaneous findings. We receive different findings for the profile of fluid temperature profile, Bejan number, velocities, and entropy generation based on these graphs, which are briefly addressed in the next paragraph.

**Figure 1:**
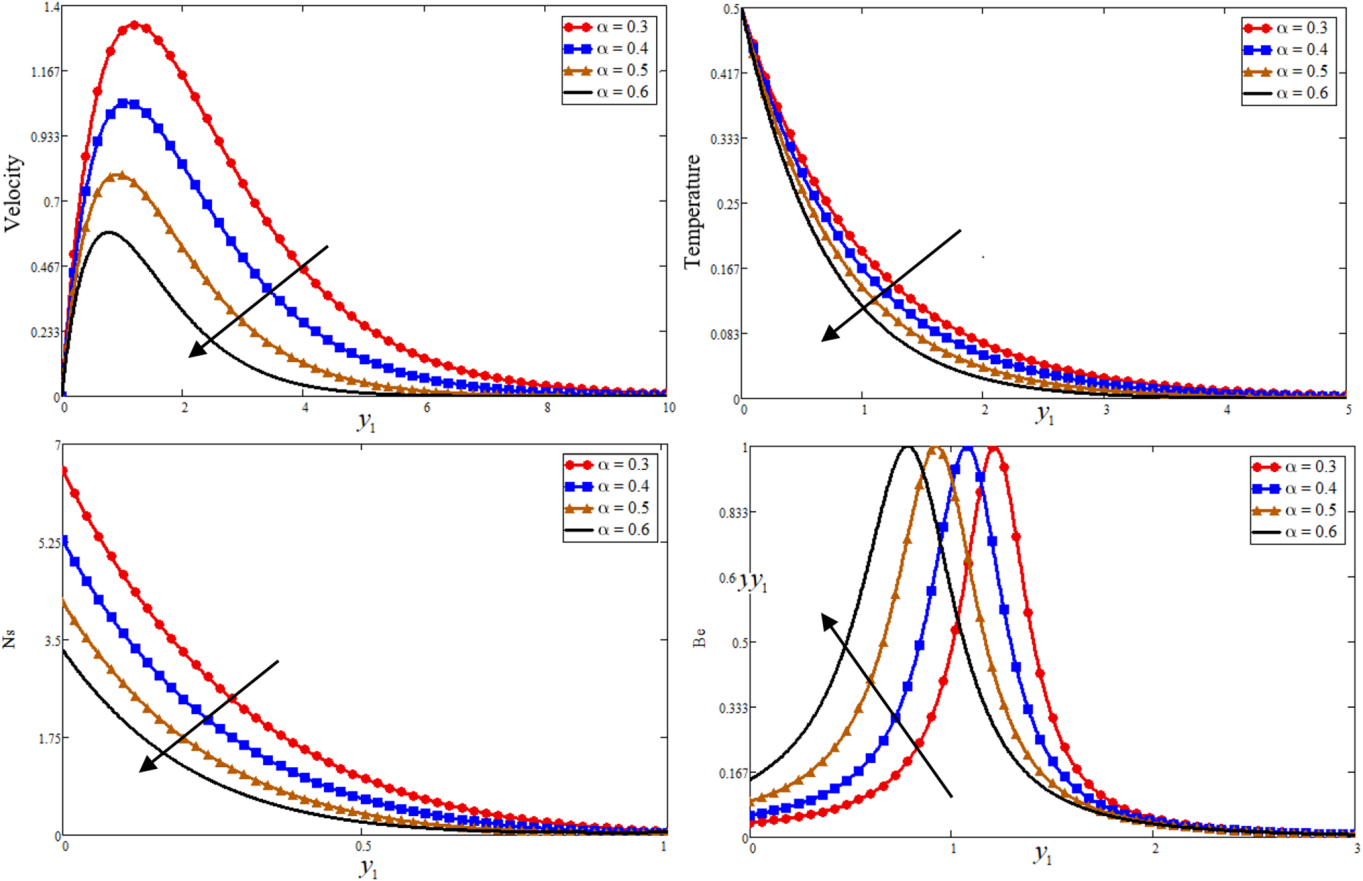
Effect of $\alpha$ Bejan number, entropy generation, temperature, and velocity.

**Figure 2:**
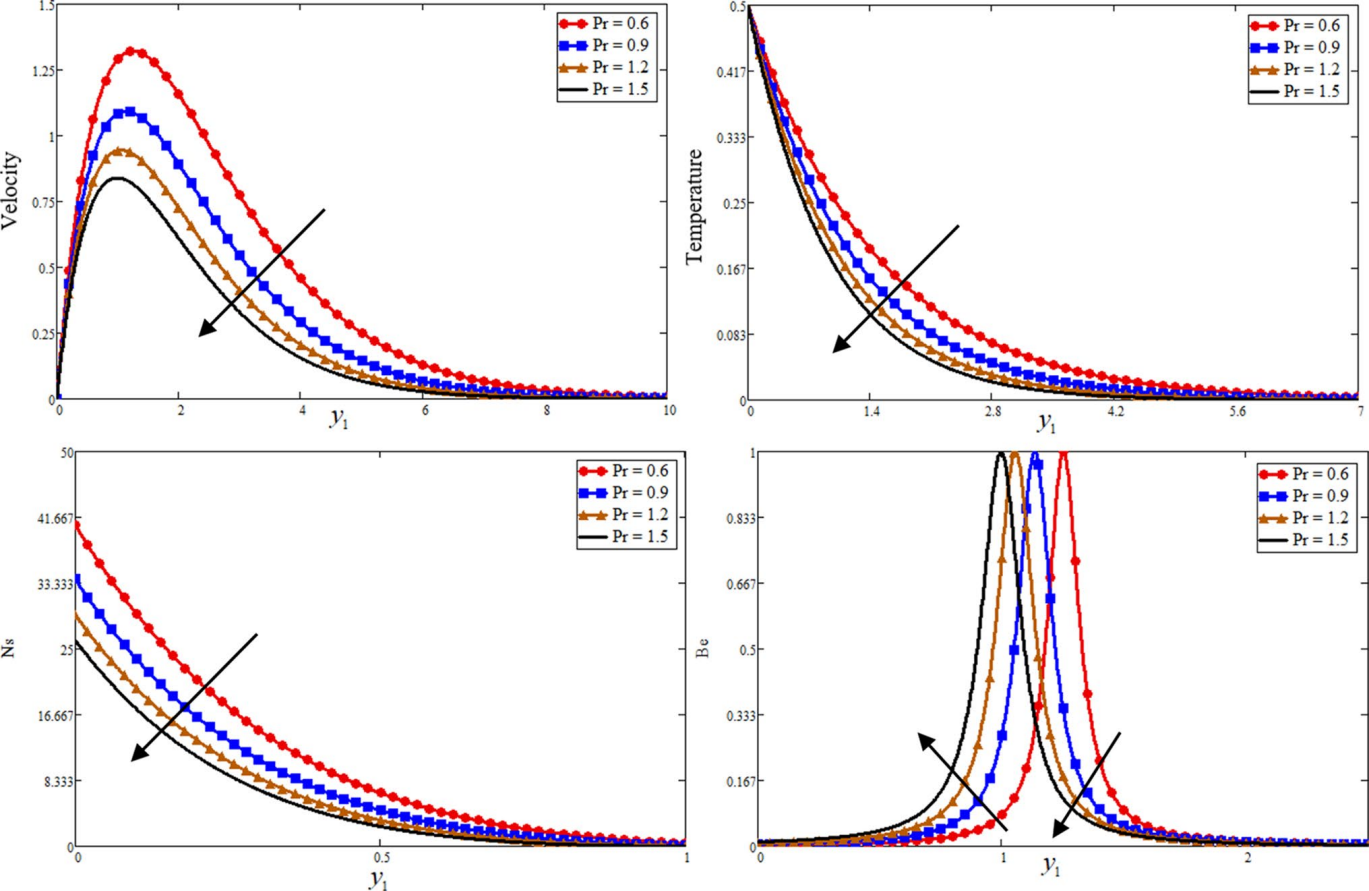
Effect of $\Pr$ Bejan number, entropy generation, temperature, and velocity.

**Figure 3:**
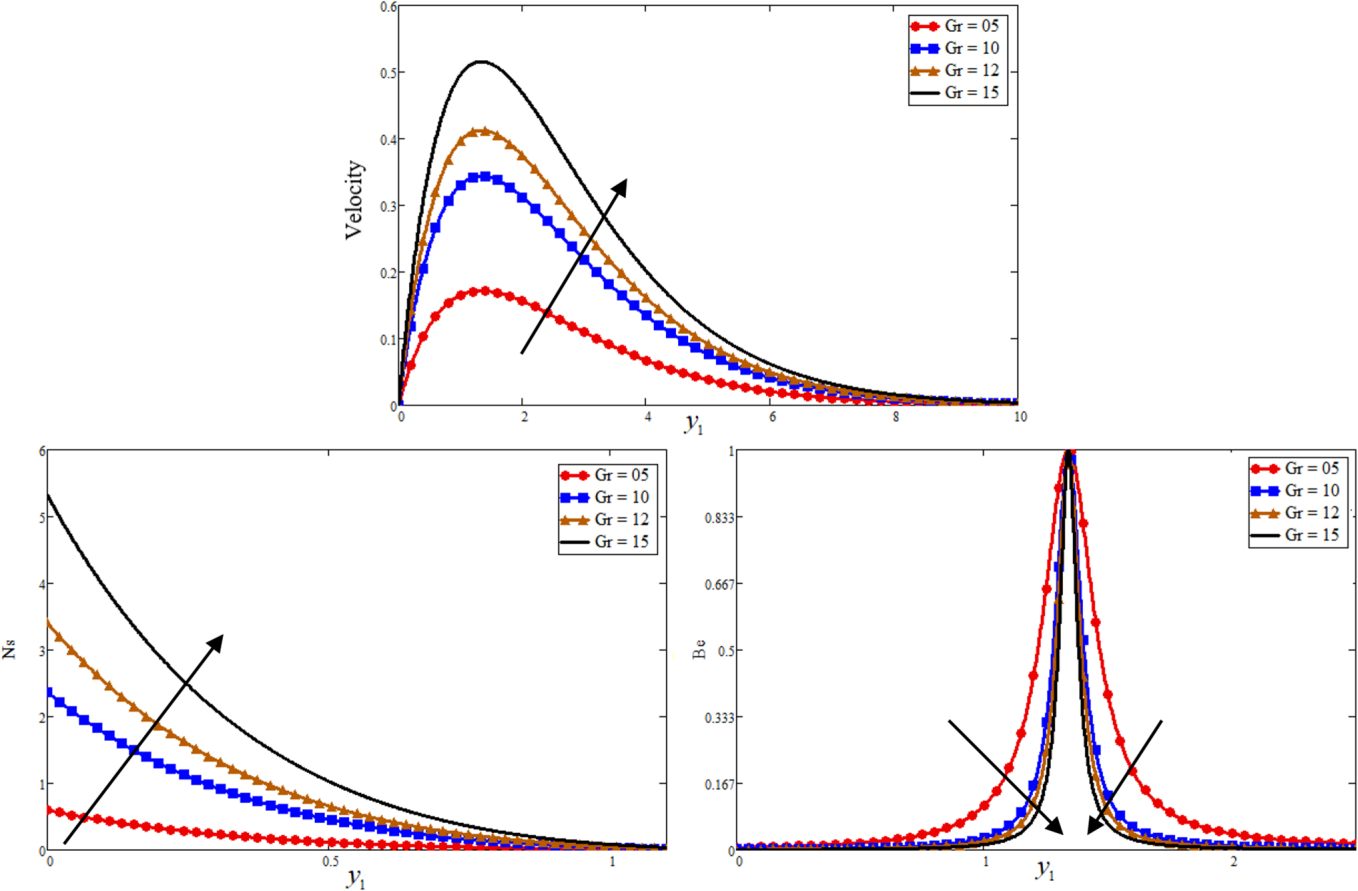
Effect of $Gr$ Bejan number, entropy generation, and velocity.

Figure 1 is reporting the graphical analysis of proportional Caputo hybrid derivative parameter $\alpha$ on velocity, temperature, entropy generation and Bejan number for less time $t_{1} = 0.5$. It is shown from the graphs that the increase of fractional parameter $\alpha$ lead to decrease the temperature, velocity of the fluid and entropy generation but increase the Bejan number near to the plate and revised after some distance. Because the thickness of heat and momentum boundary layers is inversely proportional to $\alpha$, this is the case due to which the decrease is occurs in these three profiles while due to this fact the Bejan number is increase. For the sake of justification, the o obtained results is identical to Ali et al*.*^52^ for fractional parameter. The Prandtl Number $\Pr$ is a dimensionless number that approximates the momentum-to-thermal diffusivity ratio. In heat transport and free and forced convection computations, the Prandtl Number is frequently utilized. The consequence of $\Pr$ is graphically highlighted in Fig. 2. It is clear from figure that the greater value of $\Pr$ decrease the entropy generation, temperature and velocity profile while increase the Bejan number near to the plate and far away the behaviour is revised. Physically is used in calculations of heat transfer between a moving fluid and a solid body which is equal to $\Pr = \frac{{\mu C_{p} }}{K}$. Which is clearly that $\Pr \propto \frac{1}{K}$, a greater $\Pr$ decrease in the thermal conductivity of the fluid as a result temperature profile decreases due to which a decrease is occurring in velocity and entropy generation while the Bejan number increase. In fluid dynamics and heat transfer, the Grashof number is a numerical representation of the ratio of buoyancy to a viscous force exerted on a fluid. It usually occurs in the study of natural convection situations. The consequence of $Gr$ is presented in Fig. 3. Entropy generation and velocity profiles are increasing while the decrease occurs in Bejan number with increasing of $Gr$. Physically, the fluid motion is supported by more bouncy for greater $Gr$. Due to which the above phenomena occur. Furthermore, a rise in the values of $Gr$ implies enhancement in the temperature of the plate, which leads to reduce the internal friction and intensify the gravity effects.

**Figure 4:**
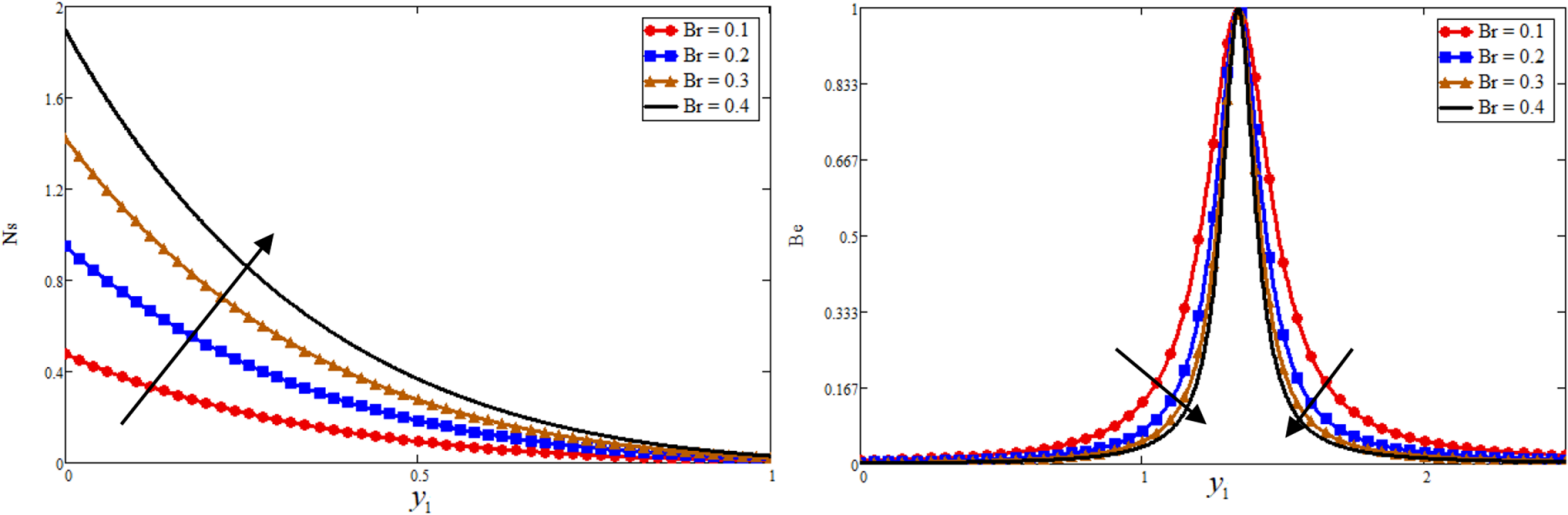
Effect of $Br$ on Bejan number, and entropy generation.

**Figure 5:**
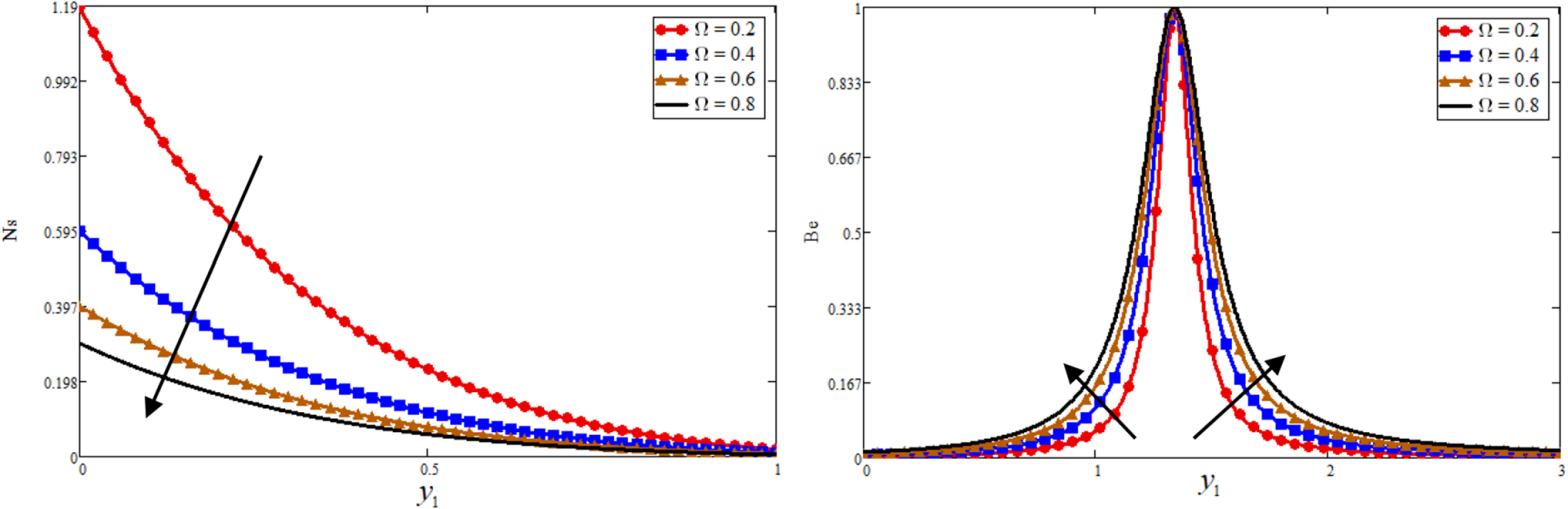
Effect of $\Omega$ on Bejan number, and entropy generation.

**Figure 6:**
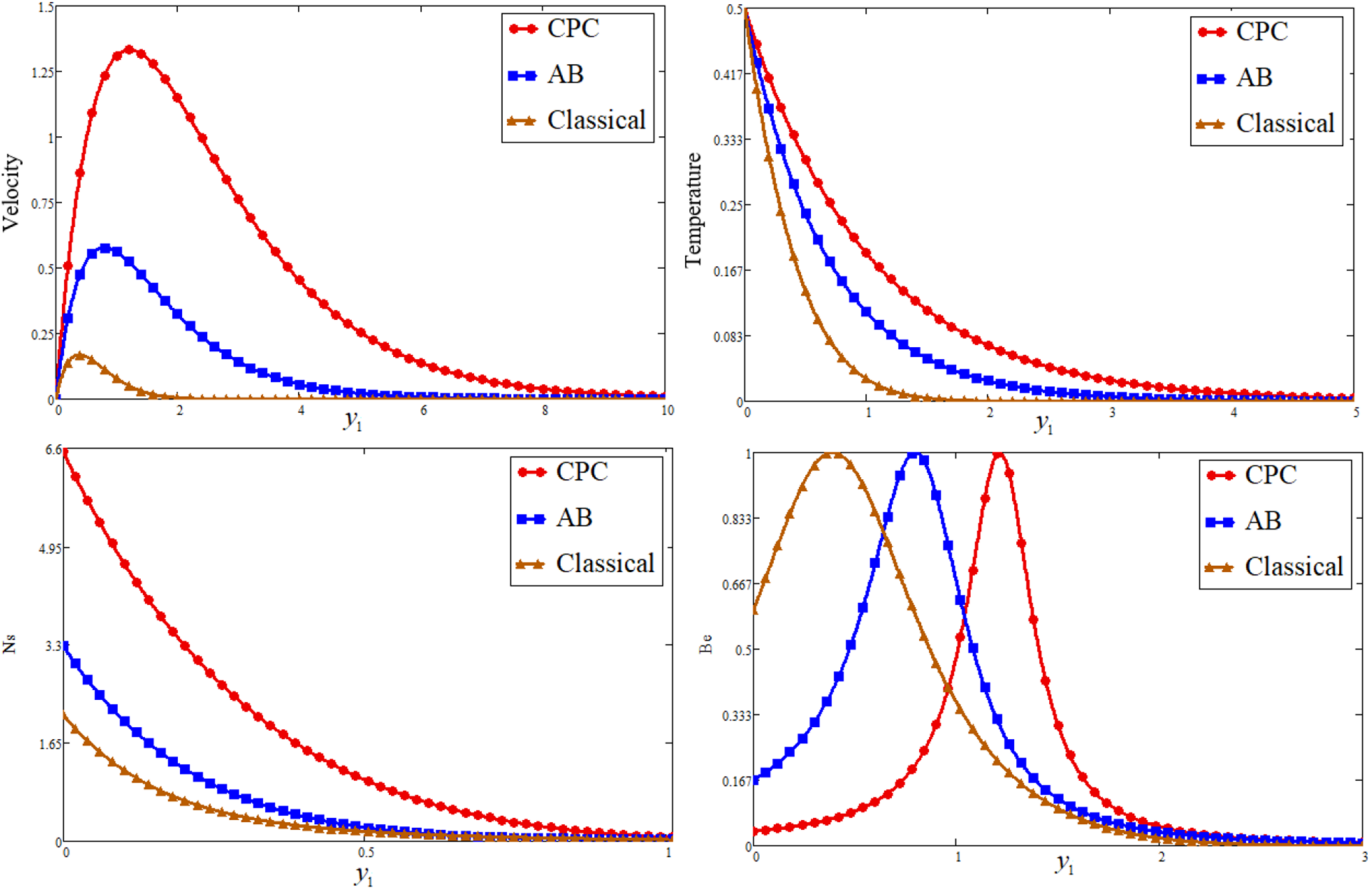
Comparative plot of Bejan number, entropy generation, temperature, and velocity.

The great quantity of heat through viscous dissipation and vice versa is generated by the Brickman number, which leads to raising the entropy generation due to the greater value of $Br$. And decrease the Bejan number, this phenomenon is highlighted in Fig. 4. The consequence of entropy generation and Bejan number against $\Omega$ is reported in Fig. 5. Physically, $\Omega$ is directly proportional to temperature difference, the greater value of $\Omega$ leads to increase the temperature difference as a result the entropy generation is decrease while due to this reason the Bejan number is increase. Figure 6 is plotted for the comparing of fractional models with classical model. The figure is plotted both newly proportional Caputo hybrid and Atangana baleanu fractional parameter $\alpha = 0.3$ and $t_{1} = 0.5$. While for both fractional models, when $\alpha \to 1$ it reduces to the classical model. It is clear for all four profiles that the proportional Caputo hybrid fractional operator gives better memory effect than Atangana baleanu fractional Operator.

## Concluding remarks

A novel report is reported of newly proportional Caputo hybrid fractional model of entropy generation for natural convection flow through vertical plate. The problem is formulated by partial differential equations with suitable boundary condition and then transform to Caputo hybrid fractional model. The generalized model is solved by applying the Laplace integral transform. The solution is compared with Atangana baleanu fractional model graphically, all graphical results are plotted via Mathcad-15 software and discussed in detail. This study's noteworthy findings are as follows:

- For $t_{1} = 0.5$, entropy generation, temperature and velocity of the fluid is reduced with the increase of $\alpha$. While Bejan number shoes the opposite behaviour.
- Proportional Caputo hybrid fractional operator have greater memory effect then Atangana baleanu fractional operator.
- $Br$ and $\Omega$ highlighted the opposite behaviour on entropy generation and Bejan number.
- The maximum range of the Bejan number is 1 occupied after some distance away from the plate.
- The ordinary fluid have less value of all four fields rather than proportional Caputo hybrid fractional and Atangana baleanu fractional derivative.

Lastly, I endorse some suggestion to the readers as bellow.

- The following idea can be extended in different geometry of calendrical coordinate.
- One of the possibility is to consider the same idea for different types of fluid with different boundary conditions.
- The idea is extended to incorporating the concentration equation with chemical reaction for many nanofluid and hybrid nanofluid.

## Data Availability

Data of this study will be made available from the corresponding author on reasonable request.
